# Beyond Neuromonitoring: Spinal Cord Perfusion, Autonomic Dysregulation, and Neurological Risk During Complex Spinal Deformity Correction

**DOI:** 10.3390/jcm15218630

**Published:** 2026-11-05

**Authors:** Michał Grabala, Piotr Kowalski, Krzysztof Zakrzewski, Jakub Kowalski, Paweł Grabala

**Affiliations:** 1 2nd Clinical Department of General and Gastroenterological Surgery, Medical University of Bialystok Clinical Hospital, ul. M. Skłodowskiej-Curie 24a, 15-276 Bialystok, Poland; michal@grabala.pl; 2 Department of Neurosurgery, Regional Specialized Hospital, ul. Dekerta 1, 66-400 Gorzow, Poland; 3 Department of Neurosurgery, Polish-Mother’s Memorial Hospital Research Institute, Rzgowska 281/289, 93-338 Lodz, Poland; krzysztof.zakrzewski@iczmp.edu.pl; 4 The Faculty of Medicine, Wroclaw Medical University, 50-556 Wroclaw, Poland; jakub.kowalski@student.umw.edu.pl

**Keywords:** intraoperative neuromonitoring (IONM), mean arterial pressure, spinal cord ischemia, spinal cord perfusion, pediatric spinal deformity, adolescent idiopathic scoliosis, neurological complications, hemodynamic optimization, perioperative management, patient safety

## Abstract

**Background:** Advances in spinal deformity surgery have expanded treatment options for pediatric and adolescent patients, but neurological complications remain among the most serious adverse events. Intraoperative neuromonitoring (IONM) enables early detection of spinal cord dysfunction, while preservation of adequate spinal cord perfusion remains an important component of perioperative neurological protection. **Objective:** This narrative review examines current evidence regarding neurological protection during complex spinal deformity surgery, with emphasis on spinal cord perfusion, IONM interpretation, hemodynamic management, and the potential interaction between mechanical and autonomic factors. An illustrative case of recurrent IONM deterioration associated with profound hemodynamic instability is included. **Methods:** A narrative review of contemporary literature was conducted, focusing on spinal cord perfusion, mechanisms of neurological injury, IONM, perioperative blood pressure management, corrective strategies, and postoperative neurological surveillance. Pediatric deformity studies were prioritized, while adult and mixed-population studies were included when relevant mechanistic or technical information was provided. The clinical case was analyzed separately from the literature synthesis. **Results:** Current evidence supports maintaining adequate systemic perfusion and promptly evaluating reversible physiological and mechanical contributors when IONM deterioration occurs. Blood pressure augmentation may restore signals in a subset of patients, but IONM changes cannot be assumed to be purely ischemic, and improved perfusion may not overcome persistent mechanical compromise. In the illustrative case, recurrent IONM signal loss occurred during corrective maneuvers and was associated with profound hypotension and bradycardia during a subsequent correction attempt. Motor and somatosensory evoked potentials were comparable to baseline by the end of surgery after release of corrective forces, hemodynamic management, and vasopressor support; they had recovered to 50% of baseline immediately before correction was resumed. Because these interventions were performed concurrently, the relative contribution of mechanical release, improved systemic perfusion, and possible autonomic mechanisms could not be determined. Motor and sensory function remained intact postoperatively, although she later reported sweating and temperature asymmetry during follow-up. **Conclusions:** Neurological protection during complex spinal deformity correction requires an integrated perioperative approach combining individualized surgical strategy, multimodal IONM, hemodynamic optimization, and postoperative surveillance. When IONM deterioration occurs, mechanical, hemodynamic, anesthetic, and technical causes should be assessed concurrently. The presented case supports consideration of a possible autonomic contribution to severe intraoperative hemodynamic instability, but does not establish a specific autonomic syndrome or causal pathway.

## 1. Introduction

Complex spinal deformity correction can place the spinal cord at risk through direct injury, altered alignment, distraction, compression, and impaired perfusion. The Scoliosis Research Society analysis of 108,419 spine procedures illustrates the clinical importance of new neurological deficits, although its heterogeneous population should not be used to estimate the risk of a particular pediatric operation [1]. More accurate instrumentation addresses one component of this risk. In a retrospective pediatric cohort, intraoperative computed tomography (CT) guidance was associated with fewer severely malpositioned screws and fewer unplanned returns to the operating room than freehand or fluoroscopic techniques [2]. These findings do not establish that technical accuracy alone prevents neurological complications during subsequent deformity correction.

Intraoperative neuromonitoring (IONM) can identify deterioration while an injury may still be reversible. In a prospective pediatric series, IONM signals recovered after mean arterial pressure (MAP) augmentation alone in some patients, supporting prompt assessment of systemic perfusion when an alert occurs [3]. Consensus guidance therefore combines hemodynamic and anesthetic assessment with evaluation of positioning, instrumentation, and the most recent surgical maneuver [4,5]. Neurological surveillance must continue after wound closure. Delayed deficits have been reported after apparently satisfactory intraoperative monitoring, and the available literature implicates several potential mechanisms, including persistent distraction, compression, and impaired perfusion [6]. Pediatric blood pressure recommendations emphasize the period before a reliable postoperative neurological examination and the need for additional surveillance after significant IONM events [7]. These recommendations are predominantly based on expert practice and observational evidence. The relationship between blood conservation and perfusion management is particularly relevant during extensive posterior reconstruction [8]. Lower pressure may reduce bleeding during exposure, while instrumentation, osteotomy, and correction introduce different neurological demands [8]. Contemporary blood management includes antifibrinolytic treatment and surgical hemostatic measures, but a reduction in blood loss should not be equated with proven prevention of spinal cord injury. A randomized trial in adolescent idiopathic scoliosis supports the efficacy of tranexamic acid for reducing bleeding [9]. The contribution of an individual measure should be distinguished from the combined effects of an entire perioperative protocol. Preoperative planning also influences the physiological demands of surgery. Angularity, rigidity, cord morphology, and the need for a three-column reconstruction may affect the choice between direct correction and a gradual or staged strategy [10]. Halo-gravity traction and temporary internal distraction have been evaluated in severe deformities, although their comparative neurological benefits remain uncertain [11,12,13,14,15]. The same caution applies to intraoperative technology: improved screw placement addresses an important source of injury but cannot establish the safety of every subsequent maneuver. A clinically useful review must therefore integrate anatomical assessment, operative decision-making, monitoring, and postoperative care rather than treat them as separate technical questions.

Autonomic cardiovascular regulation is relevant because spinal sympathetic pathways contribute to cardiac function and vascular tone, and their disruption can impair compensation for changes in circulating volume or mechanical loading [16,17,18]. During deformity surgery, potential disturbances include transient impairment of descending sympathetic control, stimulus-associated cardioinhibitory responses, and injury to regional sympathetic structures [19,20,21]. Clinical evidence remains limited: intraoperative hypotension with an inadequate chronotropic response and neuromonitoring deterioration has been reported during pediatric scoliosis surgery, while postoperative sweating and temperature abnormalities have been described after anterior and posterior deformity procedures [22,23,24]. These reports concern different operations and clinical phenotypes and do not establish a single correction-associated autonomic syndrome. They nevertheless justify considering autonomic involvement alongside mechanical, circulatory, and anesthetic explanations for cardiovascular instability. A distinction between biological plausibility and a confirmed diagnosis is therefore central to this review.

This narrative review examines neurological protection during complex spinal deformity surgery, with particular emphasis on children and adolescents. Adult and mixed-population studies are considered where they provide relevant mechanistic or technical information. A single illustrative case is used to examine the interaction between corrective maneuvers, hemodynamic instability, IONM deterioration, and possible autonomic involvement.

## 2. Methods

This narrative review was developed through targeted literature retrieval and reference-list screening. PubMed/MEDLINE records, accessible full-text repositories, and publisher websites were used to identify and verify relevant publications. The initial literature and citation update was completed on 24 September 2026. Targeted source verification and additional retrieval addressing the reviewers’ questions about autonomic mechanisms, mechanical cardiovascular compromise, and false-negative IONM outcomes were completed on 27 October 2026. The review was organized around preoperative risk assessment, spinal cord perfusion, IONM interpretation and rescue, selection of corrective strategies, postoperative surveillance, and perioperative team communication.

Targeted searches addressed perioperative blood pressure management, hypotension and bradycardia during scoliosis correction, sympathetic chain injury, and autonomic assessment. Pediatric deformity studies were prioritized. Adult cohorts, mixed populations, biomechanical studies, consensus statements, and autonomic investigations were retained when their relevance and limits of applicability could be stated explicitly.

The synthesis was based on bibliographic records, abstracts, and available full texts. References were retained only when they supported a specific statement in the text or tables. Publication identity and bibliographic details were checked against PubMed records or the publisher’s record, and linked corrections were considered. This was a narrative review rather than an exhaustive systematic review. No pooled estimates or formal risk-of-bias assessment were performed, and expert recommendations were distinguished from comparative clinical evidence.

The clinical account was considered separately from the literature synthesis. Reported events were organized by operative stage, intervention, and observed response, without reconstructing missing physiological values or assigning the effect of simultaneous interventions to a single treatment. The tables summarize the clinical sequence and relevant evidence; they are not independently validated risk scores or prescriptive treatment algorithms. The illustrative case is descriptive and cannot establish the effectiveness of an individual intervention or the cause of the observed physiological changes.

## 3. Illustrative Case

### 3.1. History and Preoperative Assessment

A 15-year-old girl with severe adolescent idiopathic scoliosis was referred to a tertiary spine deformity center because of progressive deformity, truncal imbalance, and concerns about her appearance. She had no relevant medical or neurological history, congenital anomalies, previous spinal surgery, or family history of spinal deformity. The patient and her guardians reported progression over the preceding 18 to 24 months. She remained physically active but described increasing back fatigue and psychological distress related to the deformity. She denied lower-extremity pain, weakness, sensory disturbance, gait impairment, or bowel or bladder dysfunction.

**Figure 1:**
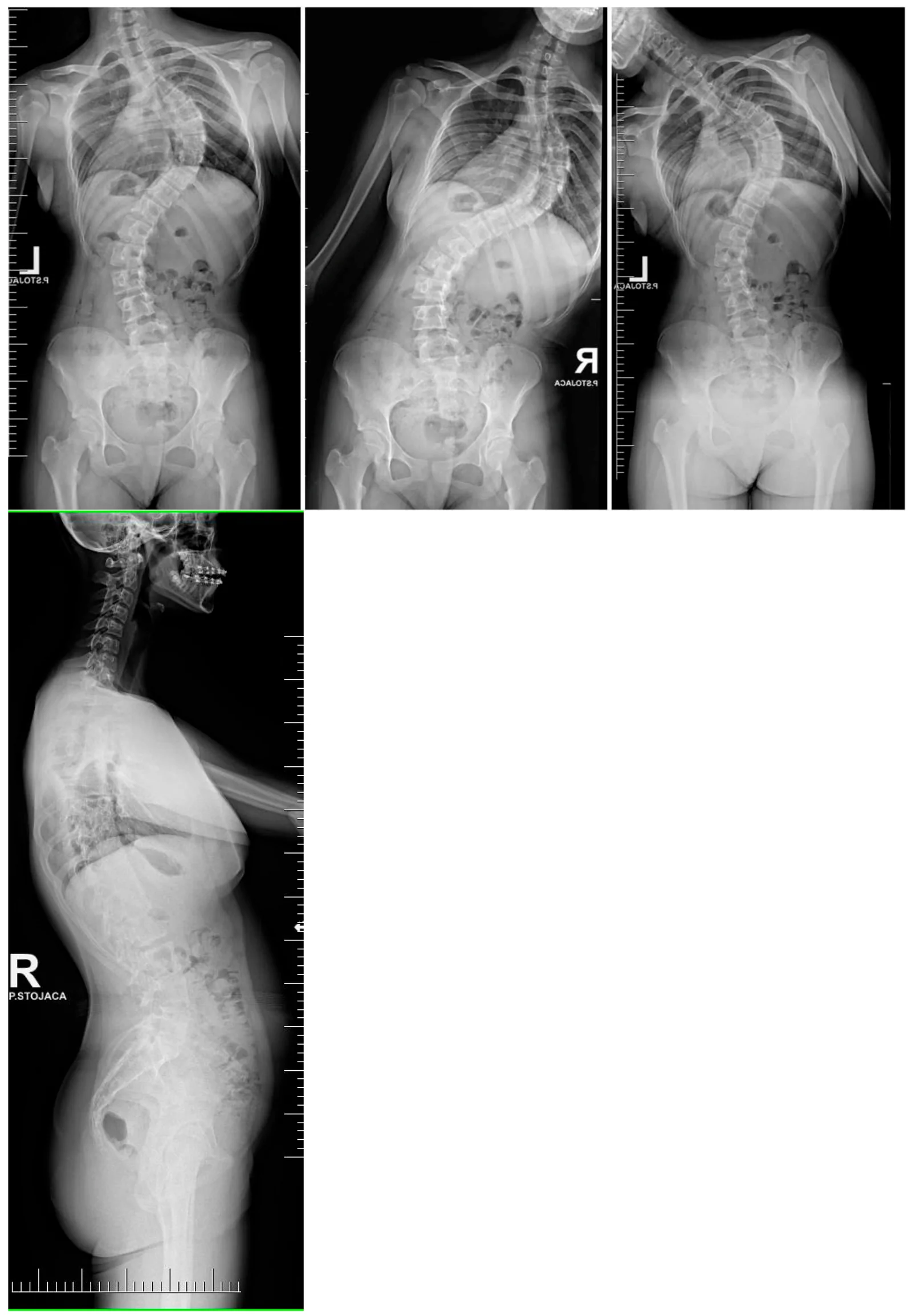
Preoperative frontal and lateral and bending radiographs illustrating the severe deformity and limited flexibility.

Physical examination revealed a prominent right thoracic rib hump, shoulder and waist asymmetry, and truncal decompensation. Muscle strength and sensation were preserved, reflexes were symmetric, and there were no clinical signs of myelopathy. Standing full-spine radiographs demonstrated a main thoracic Cobb angle of 90° and an associated lumbar Cobb angle of approximately 65°, with marked vertebral rotation and coronal and sagittal imbalance. Thoracic kyphosis measured approximately 45°, and lumbar lordosis measured 55°. Bending radiographs demonstrated limited flexibility (Figure 1). The curve pattern was classified as Lenke type 2.

Preoperative whole-spine magnetic resonance imaging (MRI) and CT were reported to show no associated intraspinal lesion or congenital osseous anomaly. Spinal cord morphology was classified as type III according to the system described by Sielatycki et al. [25], characterized by deformation or flattening of the cord against the concave apical pedicle or vertebral body without intervening cerebrospinal fluid (CSF). Following multidisciplinary assessment, posterior correction and instrumented fusion were recommended because of the severity and progression of the deformity. The patient and her family were counseled regarding the risks of neurological injury, blood loss, hemodynamic instability, infection, implant complications, pseudarthrosis, and possible revision surgery.

### 3.2. Operative Course and Neuromonitoring Events

Posterior correction and instrumented fusion were performed with multimodal intraoperative neurophysiological monitoring (IONM), including somatosensory evoked potentials (SSEPs) and transcranial motor evoked potentials (MEPs). General anesthesia was maintained using total intravenous anesthesia (TIVA) with propofol and remifentanil infusions, supplemented by a ketamine infusion and intravenous methadone administered at the beginning of surgery.

The detailed monitoring montage, including the MEP recording muscles, SSEP stimulation nerves, and use of upper-extremity control channels, could not be established from the information available for this report. These technical details are therefore not inferred from a routine institutional protocol.

Baseline MEP and SSEP responses were stable and symmetric after induction of anesthesia and prone positioning. Invasive arterial blood pressure monitoring was established before skin incision. During surgical exposure, arterial blood pressure ranged from 82/60 to 95/75 mmHg, corresponding to an estimated mean arterial pressure (MAP) of approximately 67 to 82 mmHg. The hemoglobin concentration was 15 g/dL. Instrumentation extended from T2 to L4. Ponte osteotomies at nine levels and resection of five right-sided ribs were performed to improve flexibility around the apex of the thoracic curve.

The initial correction strategy involved two 6.0-mm cobalt-chromium rods, with rod translation and segmental reduction. During the first attempt, seating the right rod using a translation maneuver resulted in loss of right-sided MEP and SSEP responses. The right rod was immediately removed, followed by an immediate return of the affected signals to baseline. Arterial blood pressure and heart rate remained stable throughout this episode. After full signal recovery, the sagittal contour of both rods was slightly reduced before a second correction attempt using rod-reduction towers. Seating the left rod produced no changes in IONM signals or hemodynamics. Subsequent seating of the right rod resulted in complete bilateral loss of MEP and SSEP responses. MAP was 80 mmHg at the time of signal loss, with no concurrent changes in arterial blood pressure or heart rate. Both rods were promptly removed, but the signals did not immediately return to baseline. Arterial blood pressure was increased to above 100/80 mmHg, with MAP exceeding 85 mmHg, to support spinal cord perfusion. MEP and SSEP responses returned to baseline bilaterally after approximately 15 min of observation.

Following full signal recovery and with stable hemodynamics, a third correction attempt was made using two 6.0-mm titanium rods prepared with less pronounced sagittal contours. The left rod was inserted first with the intention of performing rod derotation. After it was seated in the screw heads, bilateral MEP and SSEP signal loss recurred, accompanied by a decrease in arterial blood pressure to 60/50 mmHg, a fall in MAP, and the onset of bradycardia. The left rod was immediately removed, and further corrective maneuvers were suspended.

Following this third episode, even minimal contact with the chest wall was repeatedly associated with worsening hemodynamic instability, including further decreases in arterial blood pressure and MAP accompanied by bradycardia. The hemoglobin concentration at this stage was 13 g/dL. The available description does not specify the exact site, direction, or magnitude of chest wall contact or distinguish direct pressure on the ribs from transmitted pressure during handling. The temporal association therefore raised concern for a mechanically triggered cardiovascular response, potentially including an autonomic contribution, but did not establish its mechanism.

No corrective maneuvers were performed for approximately 45 min after removal of the left rod. During this interval, the anesthesiology team managed the ongoing hemodynamic instability, and vasopressor support was initiated. Intraoperative CT showed no pedicle screw malposition, cortical breach, or screw-related spinal canal compromise. Vasopressor support resulted in partial hemodynamic stabilization.

At the end of the approximately 45-min pause, MEP and SSEP responses had recovered to 50% of baseline. Correction was then resumed, and reconstruction was completed with two 6.0-mm titanium rods with reduced sagittal contouring. Gentler corrective maneuvers were performed under ongoing vasopressor support without precipitating further hemodynamic collapse. By the end of the procedure, both MEP and SSEP responses were comparable to their baseline recordings. Hemodynamic instability nevertheless persisted despite vasopressor support, with arterial blood pressure fluctuating between 80/50 and 140/80 mmHg during the final phase of the procedure. Table 1 summarizes the sequence of corrective maneuvers, IONM changes, hemodynamic events, and interventions.

### 3.3. Postoperative Course and Follow-Up

The patient was transferred while intubated to the pediatric intensive care unit (PICU) for hemodynamic and neurological monitoring. Intravenous fluids and vasopressor support were used to maintain mean arterial pressure (MAP) above 85 mmHg. Tracheal extubation was performed 24 h after surgery. Vasopressor support was required for three days, after which hemodynamic parameters normalized and support was discontinued. Subsequent examinations documented preserved motor and sensory function without new sphincter dysfunction. She remained in the PICU for four days and was then transferred to the neurosurgical ward in a hemodynamically stable condition without vasopressor support. Respiratory recovery was uncomplicated. After transfer to the neurosurgical ward, mobilization and rehabilitation progressed. The wound healed without complications, and no early implant-related or infectious complications were identified.

**Figure 2:**
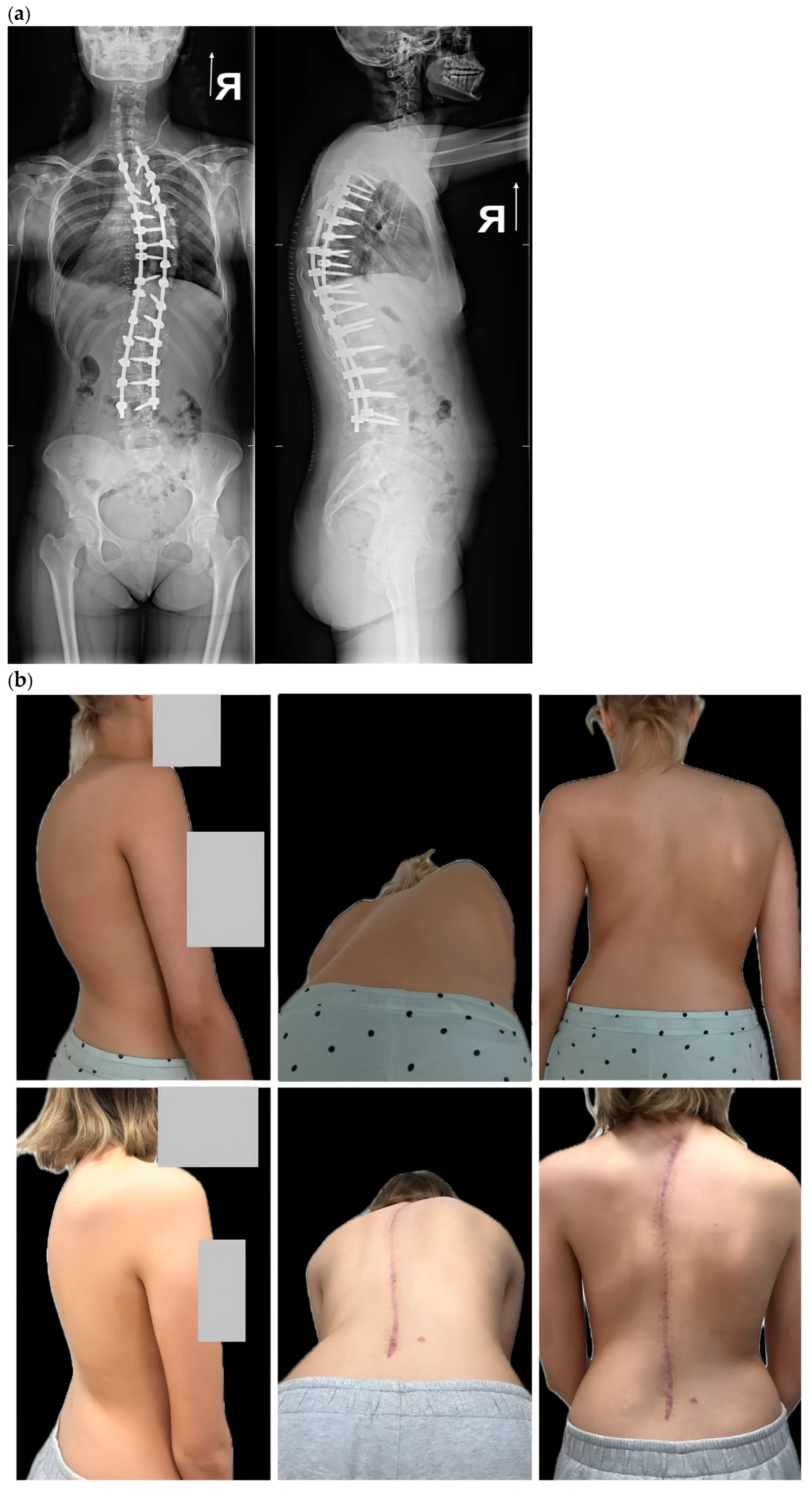
(**a**) Postoperative standing radiographs demonstrated maintained deformity correction and stable instrumentation, with thoracic and lumbar Cobb angles of 38° and 25°, respectively, and thoracic kyphosis and lumbar lordosis of 45°. (**b**) Clinical photographs before correction (upper row) and after surgery (lower row), showing lateral, forward-bending, and posterior views. The images illustrate the change in trunk alignment and rib prominence.

At the six-month follow-up, the patient had no motor or sensory deficits and had resumed normal daily activities. She reported asymmetric axillary sweating and perceived temperature differences between the lower extremities. These were patient-reported symptoms and were not confirmed by objective clinical examination or autonomic testing. The side with greater sweating, the warmer or cooler limb, the distribution within the lower extremities, and the time of onset after surgery were not established in the available account. The six-month visit identifies the time of reporting, not the onset of symptoms. Their cause and clinical significance remained uncertain, and they should not be interpreted as a confirmed autonomic deficit. Radiographs showed maintained correction and stable instrumentation. The thoracic and lumbar curves were corrected to Cobb angles of 38° and 25°, respectively (Figure 2a). Thoracic kyphosis and lumbar lordosis both measured 45°. Clinical photographs illustrate the change in trunk alignment (Figure 2b).

## 4. Discussion

### 4.1. Possible Autonomic and Mechanical Cardiovascular Mechanisms

The concurrence of profound hypotension, bradycardia, and bilateral MEP and SSEP deterioration during the third correction attempt raises the possibility of autonomic involvement, but the temporal association with manipulation does not identify the underlying mechanism. The earlier episodes occurred without hemodynamic deterioration and support sensitivity to corrective loading; they should not automatically be classified as autonomic events. Mechanical effects on the spinal cord, mechanical cardiovascular compromise, altered autonomic regulation, and anesthetic or volume-related factors may have contributed together.

The proposed autonomic explanation involves the thoracolumbar sympathetic preganglionic neurons and their descending supraspinal inputs. Disturbance of this system may reduce vascular tone, promote venous pooling, and impair cardiac sympathetic support, while preserved vagal influence may contribute to bradycardia [16]. Experimental and clinical work by Fossey et al. supports a role for impaired bulbospinal sympathetic control in reduced left ventricular contractile function after spinal cord injury [17]. Furlan et al. also associated damage to descending vasomotor pathways with cardiovascular dysfunction after cervical spinal cord injury [18]. These studies provide biological plausibility, but established spinal cord injury cannot be equated with the potentially reversible intraoperative disturbance considered here.

A stimulus-associated cardioinhibitory response is another possibility. Hernandez and Sherman described recurrent transient asystole during S1 nerve root manipulation that resolved when the stimulus stopped [20]. Dilip Chand Raja et al. reported positional bradycardia and asystole in an adolescent with neurofibromatosis and severe cervicothoracic deformity, attributing the events to vagal compression at a narrowed thoracic inlet [21]. The anatomy and operative triggers differed from those in the present case. Neither direct vagal stimulation nor a specific named reflex can therefore be inferred from bradycardia and hypotension alone.

Karsli et al. described severe hypotension, an inadequate chronotropic response, and widespread neuromonitoring deterioration during pediatric scoliosis surgery involving major blood loss and vertebral column resection [22]. The authors considered combined hypovolemia and disturbed sympathetic activity, with possible anesthetic confounding. This clinical precedent supports a broad differential diagnosis, but differences in deformity, blood loss, operative technique, and neurological outcome preclude direct equivalence with our patient.

Mechanical cardiovascular factors also deserve consideration. In the prone position, external thoracic compression or altered chest geometry may restrict venous return and cardiac filling, reducing cardiac output [26,27]. Brown et al. prospectively studied 30 adolescents undergoing scoliosis surgery and found a median 18.5% reduction in cardiac index after prone positioning, without a significant corresponding change in MAP [26]. Abcejo et al. documented pressor-refractory cardiovascular collapse in a 12-year-old patient with scoliosis; transesophageal echocardiography showed a collapsed left atrium and biventricular failure, and repositioning the chest pads restored hemodynamic stability [27]. These observations establish mechanical cardiovascular compromise as a relevant differential diagnosis, not as the demonstrated cause of the present episode.

Corrective maneuvers may also alter thoracic loading after initially tolerated positioning. In a retrospective study of 49 adolescents, Wada et al. reported seven hypotension alerts during rod attachment, rod rotation, or direct vertebral rotation in the thoracic-curve group treated with circular rods, with no alerts in the two comparison groups [28]. The authors proposed that anteriorly directed corrective forces could affect thoracic geometry and circulation. Cardiac filling and venous return were not directly measured, and the observational design cannot establish a specific mechanical pathway or a preferred rod type. Together with the positional evidence, these findings support assessing chest supports and transmitted thoracic pressure when circulatory deterioration accompanies correction [26,27,28].

In the present case, repeated deterioration during minimal chest wall contact is compatible with a mechanical cardiovascular effect, a stimulus-associated autonomic response, or both. The exact contact site and force were not characterized, and the available account contains no direct measurements of cardiac output or filling during these episodes. Stable hemodynamics during earlier stages do not exclude a later change in loading or cardiovascular tolerance. The anesthetic regimen, circulating volume, blood loss, ventilation, and rhythm should also be considered. Mechanical restriction of filling and impaired autonomic compensation may coexist, and their relative contributions cannot be determined from these observations.

The timing of cardiovascular and neurophysiological deterioration remains unresolved. Hypotension could have aggravated neural dysfunction in a cord exposed to corrective forces, but the available record does not establish whether the earliest fall in pressure or heart rate preceded, followed, or coincided with signal loss. The absence of screw malposition on CT reduces concern for an identifiable implant-related cause without excluding dynamic tension, transient compression, or regional perfusion impairment. Rod removal, reduced correction, and hemodynamic support were undertaken together; neither final signal recovery nor the preserved postoperative motor and sensory examination isolates the effect of an individual intervention.

The postoperative sweating and temperature symptoms require separate interpretation. They were reported by the patient at six months, without objective confirmation or sufficient detail to establish laterality, distribution within the lower extremities, or onset. Jagadeesh et al. described tachycardia and hyperhidrosis after posterior correction of neuromuscular scoliosis with diastematomyelia [24]. Schulte et al. assessed 24 women after anterior scoliosis correction using skin temperature measurements, plantar sweat testing, and sympathetic skin responses [23]. These studies support the possibility of postoperative autonomic manifestations and provide assessment methods, but anterior exposure carries different risks to the sympathetic chain. They do not establish a shared mechanism with the present intraoperative event.

If symptoms persist and assessment is clinically indicated, follow-up should document onset, laterality, distribution, reproducibility, and any preoperative symptoms. Thermoregulatory sweat testing evaluates central and peripheral sudomotor pathways, whereas quantitative sudomotor axon reflex testing evaluates postganglionic function; a normal postganglionic test alone does not exclude a more proximal disturbance [29]. No such results are available for this case. Terminology should remain provisional: autonomic dysreflexia involves an acute hypertensive response and does not describe these hypotensive episodes [30]; spinal shock is not a synonym for bradycardia with hypotension, and neurogenic shock cannot be assigned without sufficient evidence of its cause. We therefore describe severe hemodynamic instability with possible autonomic involvement. “Autonomic crisis,” where used in the conceptual model, denotes a hypothesis rather than an established diagnosis.

**Figure 3:**
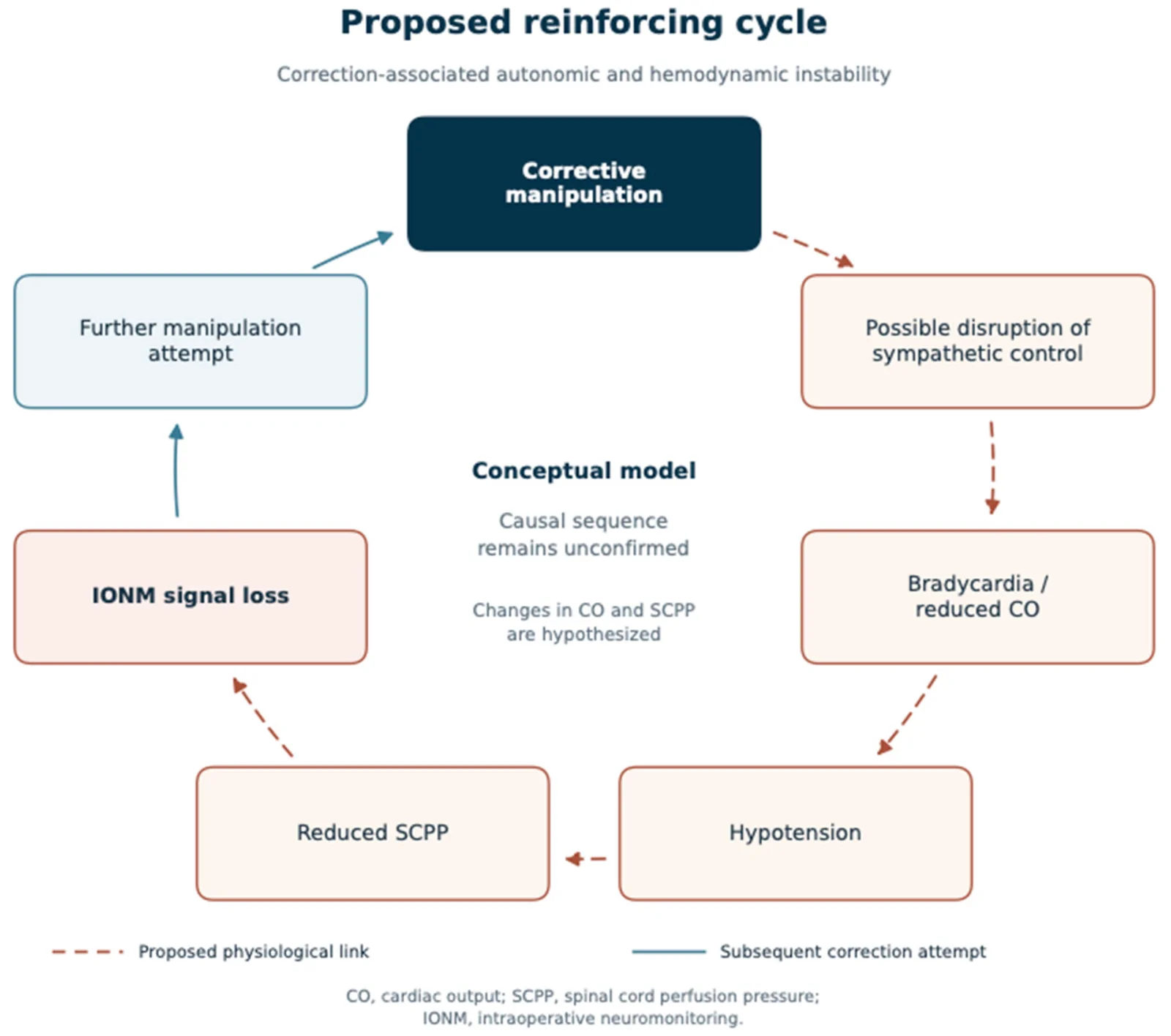
Proposed reinforcing cycle during spinal deformity correction. Corrective manipulation may disrupt sympathetic cardiovascular control, contributing to bradycardia, reduced cardiac output, hypotension, reduced spinal cord perfusion pressure, and deterioration of intraoperative neuromonitoring signals. Dashed arrows indicate hypothesized physiological relationships. Blue arrows represent potential recurrence during a subsequent correction attempt. The diagram presents one hypothesized autonomic pathway within a broader differential that also includes mechanical cardiovascular compromise from thoracic loading. It is not a reconstruction of the observed sequence; temporal and causal relationships remain unconfirmed.

Figure 3 presents a possible reinforcing interaction between corrective loading, altered autonomic cardiovascular regulation, reduced systemic perfusion, and neural dysfunction. It is one conceptual model within a broader differential that includes mechanical cardiovascular compromise. The sequence was not established by synchronized physiological measurements, and the model does not imply that autonomic dysfunction initiated the event or exclude concurrent mechanical effects. The clinical implication is concurrent assessment of surgical loading and systemic physiology, consistent with IONM rescue guidance and pediatric perfusion recommendations [3,4,5,7].

### 4.2. Spinal Cord Perfusion Throughout the Perioperative Period

Systemic arterial pressure is a readily available variable in spinal cord perfusion management, but it is not a direct measurement of regional blood flow. Interpretation of MAP must account for circulating volume, oxygenation, hemoglobin concentration, and the mechanical conditions surrounding the cord. A pressure that was tolerated during exposure may be inadequate during a later stage involving correction, blood loss, or altered cord geometry. Consensus recommendations therefore assess perfusion alongside anesthetic, technical, and surgical causes of monitoring deterioration [4,5,7]. The prospective pediatric series by Yang et al. provides clinical evidence that pressure augmentation can reverse some alerts. Among 452 procedures, 30 involved IONM changes, and signals recovered after MAP augmentation alone in six of those 30 patients [3]. This proportion applies to patients with an alert, rather than the entire operative population. Recovery in these patients supports prompt treatment of hypotension, but it does not show that all monitoring changes are ischemic or that pressure augmentation can overcome persistent mechanical compromise. Physiological observations during posterior vertebral column resection (PVCR) provide another perspective. In a prospective study of 12 patients, laser Doppler measurements demonstrated changes in spinal cord blood flow during resection and shortening [31]. The study supports an interaction between reconstruction and perfusion, but its size and operative context prevent definition of a universally safe shortening threshold. An apparently favorable response at one stage cannot be assumed to persist after further closure, translation, or definitive rod placement.

Vascular anatomy deserves particular attention when thoracic reconstruction requires segmental vessel sacrifice. In a series of 21 PVCR procedures, ligation of more segmental artery pairs was associated with greater and more prolonged SSEP amplitude changes; abnormal MEP changes and postoperative neurological deficits were not observed [32]. These findings do not establish that a particular number of ligations is safe. The absence of a clinical deficit in this small series does not establish that segmental vascular interruption is inconsequential. The distinction between systemic and regional perfusion also matters after surgery. Normal implant position and recovery of intraoperative signals do not exclude subsequent deterioration if blood pressure falls, oxygen delivery becomes inadequate, or an unresolved mechanical factor persists [6,33]. Neurological surveillance must consequently extend beyond the correction phase. Table 2 summarizes the principal anatomical, physiological, and operative considerations across the perioperative period.

### 4.3. Preoperative Assessment of Neurological Vulnerability

Preoperative assessment should establish a reproducible neurological baseline and characterize the deformity beyond its largest Cobb angle. Motor strength, sensation, reflexes, gait, and sphincter symptoms should be interpreted together with prior operations, deformity etiology, rigidity, and the proposed reconstruction. A mixed pediatric and adult institutional series found particularly frequent monitoring changes in primary neuromuscular scoliosis and revision sagittal deformity [37]. This association informs planning but does not define an individual risk probability based on diagnosis alone.

The deformity angular ratio (DAR) describes curve magnitude relative to the number of vertebral levels involved. Coronal and sagittal DARs characterize the respective planes, and total DAR combines them. Greater angularity has been associated with IONM events and neurological complications in pediatric three-column osteotomy and PVCR cohorts [34,35]. In the PVCR study by Wang et al., total DAR of at least 25 and sagittal DAR of at least 15 identified higher-risk groups [34]. These thresholds are population-specific and do not constitute universal indications for traction, osteotomy, or a fixed MAP target. Different thresholds have been reported in patients treated without vertebral column resection (VCR), emphasizing the importance of the underlying procedure and population [38]. More recent work has evaluated the deformity angular distance ratio as an additional predictor [39]. These measures may improve anatomical description, but external validation and consistent measurement are necessary before they can guide treatment selection. Their role is to supplement clinical judgment rather than replace examination and direct review of the imaging. MRI should be reviewed for both associated intraspinal abnormalities and the relationship of the cord to the concave apex. In the Sielatycki classification, type II morphology retains a symmetric or oval cord despite absent intervening cerebrospinal fluid, whereas type III includes cord flattening or deformation against the concave pedicle or vertebral body. Type III morphology was associated with greater odds of IONM loss in the original study [25]. Absence of CSF alone is therefore insufficient to classify a cord as type III.

Bakhsheshian et al. prospectively evaluated this classification in 256 pediatric and adult patients undergoing spinal deformity correction [40]. IONM loss occurred in 39.0% of patients with type III morphology, compared with 6.4% and 6.7% of those with types I and II, respectively. Type III morphology remained an independent predictor of IONM loss. Complementary evidence from the systematic review and meta-analysis by Al-Naseem et al., which included 11 studies and 3040 patients, also associated type III morphology and greater deformity angularity with IONM alerts [41]. These findings support anatomical risk assessment but do not establish an autonomic mechanism for signal deterioration.

Lee et al. developed a machine-learning-derived preoperative score that integrates spinal cord morphology, deformity angularity, neurological status, conus level, and planned upper instrumented vertebral level with other radiographic features [42]. This approach supports evaluating several interacting risk factors rather than relying on a single radiographic threshold. The score predicts cord-level IONM data loss; it does not identify the physiological cause of an individual alert or establish a safe magnitude of correction.

A retrospective study of severe adolescent scoliosis also associated smaller canal dimensions and greater convex-side canal elongation with distraction-related cord injury [43]. Because this analysis included postoperative imaging, it does not provide a prospectively validated elongation limit. Preexisting myelopathic findings, a syrinx, tethering, or congenital abnormalities require individualized assessment; their presence does not automatically indicate a particular corrective procedure. Table 3 summarizes the findings relevant to planning without assigning unvalidated categories of spinal cord reserve.

### 4.4. Preoperative Planning and Reduction in Acute Corrective Demands

Once substantial neurological or physiological vulnerability has been identified, planning should address whether the demands of definitive correction can be reduced before the principal reconstruction. Halo-gravity traction can provide gradual preoperative correction in selected severe, rigid deformities [44]. Expert consensus addresses its use and monitoring, while comparative observational studies have examined radiographic and pulmonary outcomes alongside alternative staged strategies [11,13]. These data support selective use but do not establish that traction reliably prevents neurological injury.

Temporary internal distraction is a separate technique, with different surgical requirements and potential complications. The early series by Buchowski et al. described its use as an aid to severe scoliosis correction [12]. Staged reconstruction may distribute treatment across several procedures, but the number of operations may exceed two, and reducing the burden of one procedure does not necessarily reduce the cumulative treatment burden. Planning should account for the risks of repeated anesthesia, additional instrumentation, and the interval between stages. Gradual treatment also requires active surveillance [45]. A systematic review of preoperative halo traction reported predominantly implant-related complications, together with neurological and systemic events [45]. The intended reduction in acute corrective force must therefore be weighed against traction-related risk, treatment duration, and the patient’s ability to tolerate the program. Nutritional and cardiopulmonary assessment can inform readiness for surgery, but improvement in these domains should be documented rather than assumed from the use of traction alone. In selected thoracic three-column reconstructions, vascular mapping may influence planning. An eight-patient series, with a mean age of 40 years, found that spinal angiographic findings altered the osteotomy site or neurovascular bundle preservation in three patients [46]. This adult case series does not support routine angiography before pediatric posterior fusion. It illustrates a possible role when planned vessel sacrifice and uncertain vascular anatomy create a specific concern. Table 4 summarizes these planning options and their limitations.

Skaggs et al. reported IONM changes in 9 of 22 patients undergoing correction of severe scoliosis with temporary internal distraction [50]. Signal changes reversed after release of distraction in all but one case, and no patient developed a clinical neurological deficit. These observations support the importance of monitoring during incremental loading and the potential reversibility of distraction-related changes. They do not establish that additional distraction is safe after signal recovery or that all such alerts share the same mechanism.

### 4.5. Blood Pressure Management and Blood Conservation

Blood conservation must be balanced with support of neural perfusion. A retrospective series of 327 AIS operations associated lower MAP at incision with less blood loss [51]. That observation concerns a particular operative phase and does not justify deliberate hypotension throughout instrumentation, osteotomy, or correction. The absence of reported hypotension-related complications in that cohort cannot establish safety for rare cord injuries or for patients with substantially different anatomy. Pediatric expert guidance describes phase-specific pressure goals and emphasizes agreement between the surgeon and anesthesiologist before the operation. Fletcher et al. report their practice of targeting MAP of approximately 65 to 75 mmHg during exposure and 75 to 85 mmHg during instrumentation and correction in typical adolescents, with higher goals in selected complex cases [7]. These values reflect expert practice rather than thresholds validated in comparative trials. They should not be transferred unchanged to a young child, a patient with a different baseline pressure, or a patient experiencing an active IONM alert. Pressure augmentation should be accompanied by evaluation of the cause of hypotension. Relevant factors include blood loss, circulating volume, anesthetic effects, cardiac rhythm, and the temporal relationship to the latest maneuver. Vasopressor support, volume replacement, transfusion when indicated, and modification of correction address different potential contributors. During hemorrhage, an apparently acceptable MAP should not substitute for assessment of oxygen delivery. Conversely, a monitoring change occurring immediately after a corrective maneuver requires surgical assessment while hemodynamic treatment proceeds [4,5].

Blood conservation can reduce exposure to hemorrhage and transfusion. In a randomized placebo-controlled trial involving 111 patients undergoing AIS surgery, tranexamic acid reduced intraoperative and postoperative bleeding; six placebo recipients and no tranexamic acid recipients required allogeneic transfusion [9]. No thromboembolic events or seizures were observed in that trial, but its size cannot exclude rare adverse effects. These findings support a role in blood conservation but do not directly demonstrate prevention of spinal cord injury. Device-specific evidence is less consistent. An observational AIS study reported lower blood loss with an ultrasonic bone scalpel, whereas a subsequent randomized trial did not demonstrate a reduction [52,53]. Bipolar sealing has also been evaluated as a blood-conservation adjunct [54]. The practical implication is to assess each measure on its own evidence and integrate it into a coordinated blood-management plan. A favorable effect on blood loss does not by itself establish a neurological benefit, and no single device removes the need to monitor perfusion throughout correction.

### 4.6. Intraoperative Neuromonitoring and Coordinated Rescue

Multimodal IONM provides functional information that cannot be obtained from radiographs, CT, or inspection of instrumentation alone. MEPs, SSEPs, and electromyography assess complementary aspects of neural function. Interpretation depends on reliable baseline recordings, the muscles and nerves sampled, anesthetic conditions, and technical integrity [55]. A change in a particular modality may help direct assessment, but the signal pattern does not independently identify the cause or establish the reversibility of an injury. Baseline acquisition and communication should be integrated with positioning and the anticipated critical stages of reconstruction. Comparison with the most recent reliable recording is particularly useful when an alert follows screw placement, distraction, derotation, translation, or osteotomy closure. The timing, laterality, magnitude, and distribution of the change should be communicated clearly. Focal deterioration requires assessment of the corresponding neural and peripheral structures, while generalized deterioration also raises systemic, anesthetic, and technical possibilities [4,5,55]. The response requires concurrent action. The surgeon stops the associated maneuver and assesses the need for prompt reversal while anesthesia and monitoring personnel address blood pressure, oxygenation, volume, anesthetic effects, neuromuscular blockade, electrodes, and equipment. Technical and hemodynamic checks should not delay assessment of an immediately preceding corrective maneuver. Reversal must nevertheless preserve the stability of the reconstruction. Rod removal across an unstable osteotomy is not equivalent to releasing a rod during an otherwise stable posterior fusion [4,5].

Stage-specific reports illustrate the range of surgical mechanisms. In 82 severe thoracic deformity cases treated with PVCR, monitoring events occurred during screw placement, osteotomy, gap closure, and correction [36]. A pediatric kyphosis series described rescue interventions including pressure augmentation, release of correction, correction of malalignment, additional decompression, or restoration of anterior column height [47]. The common principle is to examine what changed immediately before deterioration, including residual bone, translation, kinking, or shortening, rather than applying one maneuver to every alert. When signals fail to recover or their reliability remains uncertain, direct inspection, imaging, and a wake-up test may provide additional information when feasible [55]. Persistent unexplained deterioration may require reducing, staging, or abandoning further correction. The team must also decide how to secure the spine and obtain an early postoperative examination. Table 5 summarizes possible contributors and parallel responses; it is a clinical synthesis, not a validated diagnostic sequence. Stable IONM does not exclude every injury. Braithwaite et al. reported 16 false-negative outcomes among 5317 pediatric spinal deformity procedures [56]. Fifteen occurred with SSEP-based monitoring without transcranial MEPs (TcMEPs), whereas only one occurred with combined SSEP/TcMEP monitoring. The historical monitoring approaches included descending neurogenic evoked potentials (DNEPs), which should not be equated with TcMEPs. This distinction is important because the aggregate series should not be interpreted as the false-negative performance of contemporary SSEP/TcMEP monitoring, particularly for spinal cord pathway dysfunction. Only a minority of affected patients achieved complete recovery, reinforcing the need for a reliable postoperative examination. Modality-specific denominators and comparable patient groups are necessary to estimate relative sensitivity; multimodal monitoring does not eliminate neurological risk. In a large single-center series, Bozorgmehr et al. identified IONM alerts in 223 of 5317 pediatric spinal deformity procedures (4.2%), with intraoperative signal recovery in 156 patients [57]. Among 348 corrective actions recorded for 237 alerts, treatment of hypotension and adjustment of deformity correction were the most frequent. These findings support coordinated hemodynamic and surgical assessment during alert management. Because multiple interventions were used in an observational series, the results do not isolate the benefit of any single maneuver or establish autonomic dysfunction as the cause of hypotension.

### 4.7. Surgical Strategy and the Maximum Safe Correction

The intended benefits of deformity correction include improved balance, control of progression, preserved function, and durable reconstruction. A larger radiographic change is not a sufficient reason to continue a maneuver that is repeatedly associated with IONM deterioration or hemodynamic instability. Maximum safe correction is a decision principle rather than a preoperatively measurable angle. It describes accepting a revised alignment goal when the observed neurological response changes the balance between benefit and risk. The indication for each osteotomy should therefore be explicit. In a matched AIS series, Ponte osteotomies were associated with more critical IONM changes without an advantage in patient-reported outcomes [58]. This does not establish that posterior column osteotomies are inappropriate for every rigid curve. It does, however, challenge the assumption that additional mobilization is harmless or that greater flexibility necessarily produces a clinically meaningful benefit. Their contribution must be assessed within the complete correction strategy. More extensive osteotomies and VCR are used for selected severe angular or rigid deformities. A retrospective comparison of Schwab grade 3 to 4 and grade 5 to 6 osteotomies in congenital kyphosis and kyphoscoliosis involved substantially different baseline deformity severities [48]. Such comparisons cannot isolate the effect of osteotomy grade from the condition that required it. PVCR is a posterior approach to VCR, and these overlapping procedures do not constitute independent categories in a simple hierarchy of neurological risk. Table 6 distinguishes these overlapping surgical options and their principal demands.

In the illustrative case, recurrent alerts led to reduced corrective demands and modification of reconstruction. Preserved motor and sensory function at follow-up is favorable, but one case cannot define a generally preferred rod material, sequence, or residual curve. Table 7 sets out the potential benefits and tradeoffs of correction choices without assuming a linear relationship between each additional degree of correction and neurological injury.

### 4.8. Construct Selection, Navigation, and Robotics

Construct design should meet the mechanical requirements of the planned reconstruction. In an observational multicenter study of severe deformities, higher implant density was associated with greater coronal correction and fewer implant-related complications, while blood loss was similar between density groups [59]. These findings do not establish that lower-density constructs consistently achieve equivalent results with less blood loss. They also do not establish that maximum density is necessary in every AIS fusion. The population, anchor distribution, and intended correction remain relevant. Additional rods may share load across demanding reconstructions, although the supporting evidence requires qualification. Finite element analysis of a lumbar osteotomy construct demonstrated effects of multi-rod configurations on implant stress [60]. This is biomechanical evidence from a specific modeled reconstruction, not direct evidence of neurological protection during pediatric thoracic surgery. Construct durability and tolerance of the corrective maneuver are related clinical concerns, but they are not interchangeable outcomes.

Navigation and robotic assistance address the safety of instrumentation placement. Pediatric studies of CT navigation and robot-assisted AIS surgery support their potential to improve accuracy in the settings studied [2,61]. Accurate placement remains valuable even though it does not establish that the cord will tolerate subsequent correction. In the reported case, CT reduced concern about a screw-related cause but could not determine the effects of alignment change or systemic hypotension.

Radiation exposure should be assessed across the complete imaging pathway. A pediatric study involving robotic navigation registered to a preoperative CT reported lower intraoperative fluoroscopic exposure but a higher cumulative patient dose than the freehand protocol studied [62]. This finding is specific to the compared protocols and should not be generalized to every navigation platform. Technical accuracy, cumulative imaging exposure, and patient-specific anatomical requirements should be considered together.

### 4.9. Postoperative Neurological Protection

The transition from the operating room is a vulnerable period because continuous IONM ends while the physiological and mechanical consequences of reconstruction remain. Sedation or intubation may delay a reliable examination, and blood pressure changes may occur after an apparently satisfactory final recording. In a case series and systematic review of delayed deficits after scoliosis correction, 29 of 34 patients deteriorated within 48 h [6]. These data describe timing among selected patients with a complication, rather than incidence among all operated patients. Normal IONM and wake-up tests were explicitly documented in the three institutional cases and should not automatically be attributed to the entire literature sample. A multicenter series of 18 patients with delayed neurological changes, interpreted together with its published erratum, described peri-event hypotension in several patients and poor recovery in those with spinal cord infarction [33,]. These observations support attention to postoperative perfusion without establishing hypotension as the sole cause. The erratum corrects individual patient data and does not constitute an independent study.

The intensity and duration of observation should reflect the actual intraoperative event, ongoing support, and examination reliability. Fletcher et al. describe at least 24 h of intensive observation after transient IONM changes and longer MAP support, commonly 48 to 72 h, for patients with an established neurological deficit [7]. These are expert recommendations with limited supporting comparative data. A recovered alert, persistent signal abnormality, and established clinical deficit require different assessments and management plans. Institutional targets should be documented explicitly and reassessed as the clinical picture evolves. Postoperative hypotension is not reliably excluded by a short reassuring observation period. A recent study of 103 AIS patients found that a four-hour intensive blood pressure protocol identified only 25% of postoperative hypotensive events; no delayed neurological deficits occurred in that cohort [63]. The study concerns prediction of hypotension and cannot determine how many injuries a longer protocol would prevent. It nevertheless supports continued surveillance and clear escalation pathways after transfer. New weakness, sensory change, or sphincter dysfunction requires urgent assessment of perfusion, oxygen delivery, and potential structural causes. Blood pressure, volume, hemoglobin, and oxygenation should be assessed while the surgical team evaluates implant-related compromise, hematoma, residual compression, or another reversible factor. CT or MRI should be selected according to the clinical question and the urgency of intervention. Table 8 summarizes postoperative situations that require different levels of observation and response.

### 4.10. Multidisciplinary Organization and Continuity of Care

Standardized processes can improve consistency in preparation for and response to adverse events. Retrospective AIS studies have associated dedicated scoliosis programs and standardized operative steps with shorter operative times and reduced blood loss or resource use [65]. A later comparison reported benefits from a standardized operating room process even without a fixed dedicated team, with no significant difference in short-term complication rates between groups [66]. These findings support process reliability but do not prove prevention of rare cord injuries. Evidence from other etiologies also requires qualification [67]. In low-tone neuromuscular scoliosis, a small comparison of an integrated two-attending approach with historical controls reported shorter operations, less bleeding, and lower transfusion requirements [49]. The intervention included changes in anesthesia and blood management, so its benefits cannot be attributed to the number of surgeons alone. Similarly, improvements reported after multidisciplinary quality initiatives may reflect several concurrent changes in care [68].

Preoperative briefing should establish the planned correction, anticipated critical stages, monitoring requirements, hemodynamic goals, and responsibilities during an alert. Personnel changes during a prolonged operation should not interrupt this shared plan. After an event, documentation should connect the signal change with the latest maneuver, contemporaneous physiology, corrective actions, and the final monitoring status [4,5,7].

Handoffs should communicate unresolved concerns, examination reliability, pressure goals, blood loss and transfusion, and specific criteria for escalation. Enhanced recovery pathways may support analgesia, mobilization, and discharge after routine AIS fusion, but they do not replace individualized neurological observation after an adverse event [64]. The purpose of a common pathway is to make essential information available to the next team, while allowing management to change when the patient’s course departs from routine recovery. Table 9 summarizes these responsibilities across treatment phases.

### 4.11. Current Controversies and Limitations

The optimal intraoperative and postoperative MAP targets remain uncertain. The available evidence includes small physiological studies, retrospective cohorts, case reports, and expert recommendations across heterogeneous populations. Differences in age, diagnosis, baseline neurological function, osteotomy, anesthesia, alert criteria, and outcome definitions limit direct comparisons. A protocol developed for routine adolescent posterior fusion cannot be assumed to provide the same protection during a complex revision or thoracic VCR.

The relationship between correction magnitude and neurological risk is also incompletely defined. More severe deformities often require more extensive procedures, making it difficult to separate the contribution of the operation from that of the underlying anatomy. Absence of injury in a small series does not establish safety, while a monitoring alert that resolves after intervention does not by itself identify the mechanism or quantify the injury that was prevented.

This narrative review was not systematic and did not include formal risk-of-bias assessment or pooled effect estimates. The tables summarize evidence and clinical considerations rather than provide validated risk scores or treatment algorithms. The presented case account lacks synchronized MAP, heart rate, IONM, anesthetic, and intervention data. Approximately 45 min of stabilization should not be interpreted as 45 min of continuous hypotension or complete signal loss. The detailed monitoring montage and the exact chest wall manipulations could not be established from the information available for this report. Simultaneous interventions prevent attribution of recovery to a single treatment, and the late symptoms are patient-reported, without objective confirmation or an established onset and laterality.

### 4.12. Future Directions

Prospective multicenter studies should link anatomical measurements with time-stamped physiology, operative maneuvers, and IONM events. Candidate predictors such as DAR, cord morphology, and canal dimensions require external validation and assessment of whether they improve decisions beyond established clinical evaluation. Models should distinguish prediction of an alert from prediction of persistent neurological disability, because these outcomes have different implications.

Studies of individualized perfusion management should report baseline blood pressure, age, operative phase, the depth and duration of hypotension, and the interventions used. Postoperative outcomes should include the timing and reliability of neurological examinations rather than discharge status alone. Consistent definitions would facilitate comparison across institutions and help evaluate management of uncommon delayed deficits.

Direct assessment of cord blood flow or oxygenation may complement electrophysiological monitoring, but the small physiological studies available do not establish a routine clinical role [31]. Computational or machine-learning approaches to combined imaging and physiological data would similarly require prospective validation before influencing care. These are research directions, not established protective strategies. More structured follow-up of sweating, temperature, and other autonomic symptoms could also clarify whether postoperative dysfunction is regional, transient, preexisting, or related to the intraoperative event.

## 5. Conclusions

Neurological protection during complex spinal deformity correction depends on the interaction between patient-specific anatomy, the mechanical demands of reconstruction, and perioperative physiological support. Accurate instrumentation is essential, while preoperative assessment, individualized correction, multimodal IONM, blood pressure management, and postoperative examination address additional sources of risk. An IONM alert should prompt concurrent surgical, hemodynamic, anesthetic, and technical assessment. When deterioration follows a corrective maneuver, the maneuver should be stopped and the need for reversal assessed promptly, with attention to spinal stability. Persistent uncertainty may require reduced correction, staged treatment, or abandonment of further corrective steps. The aim is a durable reconstruction that preserves neurological function, with radiographic goals adjusted to the observed physiological response.

The illustrative case demonstrates recurrent, potentially reversible dysfunction accompanied by profound hypotension and bradycardia. Motor and sensory function were intact at six months, while she reported sweating and temperature asymmetry during follow-up. The observations support a possible autonomic contribution but do not confirm a specific autonomic syndrome, sympathetic chain injury, or the relative effects of the simultaneous rescue interventions.

Current evidence supports a continuous perioperative approach while leaving optimal pressure targets and individual correction limits unresolved. Better prospective data, explicit reporting of IONM events, and reliable follow-up are needed to refine these decisions without overstating the certainty provided by existing classifications or institutional protocols.

## Figures and Tables

**Table 1:** Sequence of intraoperative events in the illustrative case.

Operative Stage	IONM and Hemodynamic Findings	Intervention and Response
First correction attempt: right cobalt-chromium rod	Right-sided MEP and SSEP loss after seating the right rod by translation. Arterial blood pressure and heart rate remained stable.	Removal of the right rod was followed by an immediate return of the affected signals to baseline.
Second correction attempt: cobalt-chromium rods with reduced contouring	Left rod seating caused no changes. Right rod seating produced complete bilateral MEP and SSEP loss at a MAP of 80 mmHg, without concurrent changes in arterial blood pressure or heart rate.	Both rods were removed. Arterial blood pressure was increased to above 100/80 mmHg, with MAP exceeding 85 mmHg. Signals returned to baseline after approximately 15 min.
Third correction attempt: left titanium rod	Bilateral MEP and SSEP loss recurred after seating the left rod intended for derotation. Arterial blood pressure fell to 60/50 mmHg, with a decrease in MAP and onset of bradycardia.	The left rod was immediately removed, and further corrective maneuvers were suspended.
Contact with the chest wall following the third episode	Minimal contact was repeatedly associated with worsening hypotension and bradycardia. Hemoglobin was 13 g/dL.	Hemodynamic management continued. Mechanical cardiovascular and autonomic contributions remained possible; neither was established.
Approximately 45-min pause in corrective maneuvers	Hemodynamic instability persisted. Intraoperative CT showed no screw malposition, cortical breach, or screw-related canal compromise. MEP and SSEP responses had recovered to 50% of baseline immediately before correction was resumed.	No corrective maneuvers were performed. Vasopressor support was initiated and produced partial hemodynamic stabilization.
Completion of reconstruction	MEP and SSEP responses improved from 50% of baseline before correction resumed to levels comparable to baseline by the end of surgery. Arterial blood pressure fluctuated between 80/50 and 140/80 mmHg despite vasopressor support.	Reconstruction was completed using two 6.0-mm titanium rods with reduced contouring and gentler correction, without further hemodynamic collapse.

The approximately 45-min interval denotes a pause in corrective maneuvers and does not specify the duration of continuous hypotension or complete IONM signal loss. MEP and SSEP responses had recovered to 50% of baseline immediately before correction was resumed. The table summarizes the operative sequence and is not a time-synchronized anesthetic and IONM record. CT, computed tomography; IONM, intraoperative neuromonitoring; MAP, mean arterial pressure; MEP, motor evoked potential; SSEP, somatosensory evoked potential.

**Table 2:** Determinants of spinal cord perfusion and function across treatment phases.

Phase or Domain	Relevant Findings	Clinical Implication
Preoperative anatomy	Angular deformity, rigidity, concave apical cord contact, reduced CSF space, and associated intraspinal abnormalities.	Review the demands of planned correction; neither cord morphology nor DAR defines an individual perfusion threshold [25,34,35].
Preoperative physiology	Baseline blood pressure, anemia, cardiopulmonary status, and anticipated blood loss.	Establish an individualized pressure and blood-management plan before surgery [5,7].
Exposure and instrumentation	Anesthetic effects, position, bleeding, arterial pressure, and implant placement.	Interpret pressure by operative phase and reassess signals after relevant changes [4,5,7].
Correction and osteotomy	Distraction, translation, derotation, closure, shortening, and segmental vascular interruption.	Mechanical and vascular contributors may coexist; normal systemic pressure does not exclude regional compromise [31,32,36].
Systemic oxygen delivery	Hemoglobin, oxygenation, circulating volume, and ongoing blood loss.	Treat the contributing physiological disturbance while assessing the latest surgical maneuver [4,5].
Early postoperative period	Pressure fluctuations, anemia, sedation, examination reliability, and any residual mechanical concern.	Continue observation and investigate new findings despite recovered intraoperative signals [6,7,33,].

Author synthesis. No quantitative spinal cord reserve or universal perfusion threshold is assigned. CSF, cerebrospinal fluid; DAR, deformity angular ratio; MAP, mean arterial pressure.

**Table 3:** Preoperative findings that inform neurological risk assessment.

Factor	Assessment	Evidence and Limitation	Implication for Planning
Baseline function	Motor, sensory, reflex, gait, and sphincter findings; prior surgery and deformity etiology.	Risk varies by diagnosis and operative history; cohort associations do not assign individual probabilities [37].	Record a reproducible baseline and identify findings requiring further assessment.
Associated neural abnormality	Preexisting deficit, myelopathic findings, a syrinx, tethering, or another intraspinal lesion.	Interpret the neurological examination and imaging together; a diagnostic label alone does not quantify operative risk [5].	Clarify the lesion, obtain relevant specialist assessment, and individualize the reconstruction.
Cord morphology	Axial T2 MRI at the curve apex and associated intraspinal abnormalities.	Type III morphology includes cord deformation or flattening without intervening CSF. Absent CSF alone is insufficient [25].	Review the concave apex and discuss the anticipated demands of correction.
Angular severity	Coronal, sagittal, and total DAR using a stated measurement method.	Associations and thresholds differ between PVCR, other three-column osteotomy, and non-VCR cohorts [34,35,38].	Use angularity with other findings; avoid a universal DAR-based treatment rule.
Additional imaging measures	DADR and canal dimensions where relevant imaging is available.	Recent observational findings require external validation; postoperative elongation does not define a prospective safe limit [39,43].	Interpret as additional information rather than established decision thresholds.
Physiological and operative demands	Baseline blood pressure, anemia, cardiopulmonary status, rigidity, planned osteotomy, and anticipated blood loss.	The contribution of each factor depends on the patient and procedure [5,7].	Agree on hemodynamic goals, monitoring, rescue options, and postoperative level of care.

Author synthesis of the cited evidence. This table is not a validated risk score and does not assign measurable categories of spinal cord reserve. CSF, cerebrospinal fluid; DADR, deformity angular distance ratio; DAR, deformity angular ratio; MRI, magnetic resonance imaging; PVCR, posterior vertebral column resection.

**Table 4:** Patient-specific planning considerations before definitive correction.

Clinical Setting	Options to Consider	Limitations and Questions to Resolve
Flexible AIS with preserved function	Posterior fusion with maneuvers selected for the actual curve and balance objectives.	A reassuring baseline does not remove the need for multimodal monitoring and a rescue plan [4,5].
Severe rigid AIS	Halo-gravity traction, temporary internal distraction, or staged correction when appropriate.	These are different strategies. Evidence does not establish a universal neurological advantage; feasibility and treatment burden differ [11,12,13].
Angular congenital deformity or severe kyphosis	Tailored osteotomy or resection according to rigidity, location, neural findings, and required reconstruction.	More extensive procedures are often used for more severe anatomy; procedure grade and baseline risk are confounded [34,47,48].
Type III cord morphology or neurological abnormality	Detailed MRI review, explicit correction goals, and preparation to reduce or stage correction.	Neither the MRI category nor the diagnosis dictates one procedure or a measurable reserve category [5,25].
Revision deformity	Review prior surgery, fused segments, available anchors, imaging needs, and stability during reconstruction.	Risk depends on the actual revision; navigation assists placement but cannot establish correction tolerance [2,5,37].
Neuromuscular deformity with physiological comorbidity	Coordinate surgical, anesthesia, blood-management, respiratory, and postoperative plans.	Evidence from integrated programs cannot isolate the effect of any single component [37,49].
Thoracic three-column surgery with planned vessel sacrifice	Consider whether vascular mapping would answer a specific anatomical concern.	Evidence for spinal angiography includes a small adult series, not a recommendation for routine pediatric screening [46].
Substantial anticipated blood loss or poor physiological condition	Optimize identified reversible problems, plan blood availability, and assess timing or staging.	The expected benefit must be balanced against additional procedures and treatment duration; do not assume optimization from traction alone [5,7,9].

Author synthesis, not a validated algorithm. Choices require direct clinical and imaging assessment. No operation is assigned automatically from diagnosis, Cobb angle, or MRI category. AIS, adolescent idiopathic scoliosis; MRI, magnetic resonance imaging.

**Table 5:** Possible causes of IONM deterioration and coordinated responses.

Possible Contributor	Clinical Context	Concurrent Actions
Impaired systemic perfusion	Signal deterioration with hypotension, blood loss, or impaired oxygen delivery.	Restore appropriate blood pressure; assess volume, hemoglobin, and oxygenation while evaluating the latest surgical maneuver.
Correction-related mechanical stress	Change during distraction, translation, derotation, compression, or osteotomy closure.	Stop the maneuver and assess prompt reversal. Maintain spinal stability and evaluate alignment, cord tension, and residual compression.
Implant or root compromise	Focal or unilateral change associated with instrumentation; corresponding EMG activity may be present.	Inspect or image the relevant region and revise the suspected cause when indicated. A signal pattern alone does not diagnose malposition.
Nerve-root irritation	Focal EMG activity or myotomal MEP change after retraction or instrumentation.	Evaluate the corresponding root and implant, release the suspected mechanical contributor when indicated, and reassess.
Osteotomy-related compression or instability	Change during resection, closure, or reconstruction.	Assess bone fragments, kinking, translation, shortening, and the need for decompression or restoration of anterior column height.
Position or peripheral compression	Upper-extremity or focal changes after positioning or limb manipulation.	Check limb position, shoulder traction, pressure points, and peripheral perfusion while excluding a central or systemic cause.
Anesthetic or technical factors	Changes after anesthetic dosing, neuromuscular blockade, electrode displacement, or equipment disturbance.	Review anesthetic changes and neuromuscular function, check connections and electrodes, and repeat acquisition without delaying other necessary actions.
Temperature disturbance	Signal deterioration in the setting of a change in core temperature.	Check temperature and correct relevant hypothermia while evaluating other physiological and operative contributors.
Persistent unexplained deterioration	Signals remain abnormal after initial assessment and intervention.	Reassess all contributing factors, consider imaging, direct inspection or a wake-up test when feasible, and determine whether correction should be reduced or discontinued.

Adapted as an author synthesis from consensus guidance and deformity-specific clinical reports [4,5,36,47,55]. Actions are concurrent rather than a rigid sequential checklist. The pattern of signal loss is suggestive, not diagnostic. Maneuver reversal must account for the stage and stability of reconstruction. EMG, electromyography; IONM, intraoperative neuromonitoring.

**Table 6:** Corrective procedures and adjuncts for severe deformity.

Strategy	Potential Role	Limitations and Neurological Considerations
Posterior fusion with tailored correction	Definitive reconstruction using maneuvers selected for the deformity and patient.	Fusion is the overall procedure; osteotomies may be components of it. Safe implant placement does not establish tolerance of subsequent correction [2,5].
Posterior column osteotomies	Additional mobilization when a specific correction objective requires it.	Routine use in AIS is not supported by every comparative study. The potential gain must be weighed against additional operative demands and IONM events [58].
Pedicle subtraction or other selected three-column osteotomy	Correction of a fixed angular or sagittal deformity when the required change is appropriate for the selected osteotomy.	Requires planning for bleeding, stability, neural decompression, and closure. Comparative outcomes depend on baseline severity and osteotomy grade [48].
Vertebral column resection, including PVCR	Extensive mobilization and reconstruction in selected severe rigid or angular deformities.	PVCR is a posterior VCR approach. Resection, closure, translation, vascular interruption, and reconstruction create different monitoring risks [32,34,36].
Halo-gravity traction	Gradual preoperative correction in selected severe deformities.	Requires monitoring and pin care; implant-related and neurological complications can occur. Consensus practice does not prove superiority over other strategies [11,13,14,45].
Temporary internal distraction and staged correction	Progressive correction before definitive fusion in selected rigid curves.	May require multiple operations. Evidence is mainly observational and does not establish a universal reduction in neurological risk [12,13].
Tailored implant density and rod configuration	Control of correction and durability of reconstruction.	Higher density has shown advantages in severe-deformity observational data; results should not be generalized to all patients. Multi-rod biomechanical findings do not directly establish neurological benefit [59,60].
Navigation or robotic assistance	Assistance with instrumentation in selected anatomy and workflows.	Accuracy and cumulative radiation exposure depend on technique and imaging protocol. These technologies do not replace IONM or perfusion management [2,61,62].

These options overlap and are not a hierarchy of neurological risk or a validated selection algorithm. The appropriate extent of correction depends on anatomy, physiological tolerance, reconstruction requirements, and the clinical objective. AIS, adolescent idiopathic scoliosis; IONM, intraoperative neuromonitoring; PVCR, posterior vertebral column resection; VCR, vertebral column resection.

**Table 7:** Balancing the intended benefit of correction against operative tradeoffs.

Decision	Potential Benefit	Tradeoff and Basis for Reassessment
Additional coronal or sagittal correction	Improved alignment and balance within the operative objective.	Further distraction, translation, or angular change may provoke deterioration; the response must be assessed during the actual maneuver [5,36,47].
Additional posterior column release	Greater flexibility for a defined correction objective.	A matched AIS study reported more critical IONM changes without a patient-reported outcome advantage; routine benefit cannot be assumed [58].
More extensive three-column reconstruction	Ability to address a severe fixed deformity and reconstruct alignment.	Bleeding, neural risk, and instability must be considered together with the anatomy that prompted the procedure [34,36,48].
Greater implant density	More anchors for correction and load distribution.	Severe-deformity observational findings favor greater coronal correction and fewer implant complications, but do not define the ideal density for every patient [59].
Additional rods	Potential reduction in implant stress in a demanding reconstruction.	Biomechanical evidence is construct-specific and does not prove neurological protection in pediatric correction [60].
Traction or staged reconstruction	Opportunity for gradual correction before definitive fusion.	Longer treatment, additional procedures, and traction-related complications must be weighed against the anticipated operative benefit [11,12,45].
Reduced or discontinued correction after an alert	Opportunity to address the trigger and preserve neurological function.	Accept residual deformity when necessary while ensuring a stable reconstruction and an explicit postoperative plan [4,5,47].

These tradeoffs are a clinical synthesis, not a quantitative relationship between correction magnitude and neurological risk. Maximum safe correction is an intraoperative decision principle, not a validated radiographic threshold. AIS, adolescent idiopathic scoliosis; IONM, intraoperative neuromonitoring.

**Table 8:** Postoperative neurological surveillance and escalation.

Situation	Assessment and Response	Evidence or Limitation
Recovered intraoperative alert	Communicate the event and final signals; obtain serial examinations and maintain the individualized pressure plan.	Expert guidance describes at least 24 h of intensive observation, but duration is not established by comparative trials [7].
Persistent signal abnormality or new clinical deficit	Arrange intensive monitoring, neurological reassessment, physiological support, and urgent surgical evaluation.	Longer support, often 48 to 72 h in expert practice, is distinct from management of a fully recovered alert [7].
Examination limited by sedation or intubation	Document the limitation, continue appropriate monitoring, and obtain a reliable examination as soon as clinically feasible.	A normal final IONM record cannot replace the postoperative examination [7,56].
Hypotension without an identified new deficit	Assess pressure trend, blood loss, volume, hemoglobin, oxygenation, and medication effects; escalate if persistent.	A short period of intensive monitoring does not reliably predict all later hypotensive events [63].
New weakness, sensory change, or sphincter dysfunction	Evaluate physiological and structural causes concurrently; select urgent CT or MRI and intervention according to the suspected cause.	Delayed deficits may follow an initially reassuring course; absence of screw malposition does not exclude other mechanisms [6,33,].
Transfer to a ward or another team	Provide pressure goals, examination findings, alert history, ongoing support, and explicit escalation criteria.	Observation and response capacity must match the patient’s needs; an ERAS pathway does not supersede an adverse-event plan [7,64].
Persistent sweating or temperature asymmetry	Record onset, distribution, laterality, preoperative symptoms, and objective findings; consider specialist assessment when indicated.	Symptoms alone do not localize a sympathetic lesion or prove the cause of the intraoperative event [23,29].

Author synthesis. Actual pressure targets, duration of support, and level of care require individual clinical assessment. The durations cited describe expert recommendations, not universal standards. CT, computed tomography; ERAS, enhanced recovery after surgery; IONM, intraoperative neuromonitoring; MRI, magnetic resonance imaging.

**Table 9:** Multidisciplinary responsibilities across the perioperative period.

Phase	Principal Responsibilities	Information to Document and Communicate
Preoperative	Review neurological and imaging findings, anticipated correction, blood loss, and the need for staged treatment.	Baseline function and pressure; patient-specific concerns; planned IONM modalities, hemodynamic goals, and rescue options [5,7].
Preoperative preparation	Assess readiness, blood availability, the need for traction or staging, and postoperative support.	Reason for the chosen strategy; identified reversible concerns; monitoring and escalation arrangements [5,7,11].
Positioning and instrumentation	Establish reliable signals and pressure monitoring; assess positioning and instrumentation-related changes.	Baseline signal quality, relevant anesthetic conditions, positioning events, and implant concerns [4,5].
Correction and reconstruction	Relate IONM changes to maneuvers and physiology; respond concurrently and preserve mechanical stability.	Time, side, modality and magnitude of the alert; MAP and heart rate; interventions and response; final alignment and signal status [4,5].
Three-column reconstruction	Coordinate resection, closure, temporary stabilization, and monitoring at critical steps.	Operative stage, cord geometry, vascular interruption when relevant, blood loss, and response to any alert [32,36,47].
Completion of reconstruction	Confirm final signal status, alignment, stability, and any unresolved concern.	Accepted residual correction, examination plan, pressure goals, and need for additional intervention [4,5].
Transfer and early postoperative care	Obtain a reliable examination and monitor perfusion; investigate new deficits promptly.	Final IONM status, examination reliability, individualized blood pressure plan, blood loss, transfusion, and escalation criteria [6,7,33,].
Recovery and follow-up	Continue neurological assessment, rehabilitation, and review of persistent symptoms.	Motor, sensory and sphincter findings; functional recovery; onset and distribution of sweating or temperature symptoms when present [29,64].

Author synthesis. Monitoring intensity and duration should reflect actual events, ongoing support requirements, and examination reliability. A transient alert, a persistent signal abnormality, and an established deficit are different clinical situations. IONM, intraoperative neuromonitoring; MAP, mean arterial pressure.

## Data Availability

No new data were created or analyzed in this study. Data sharing is not applicable to this article. The evidence discussed is available in the cited publications.
